# A mycotic aneurysm of a jejunal branch treated by aneurysmectomy without bowel resection

**DOI:** 10.1016/j.jvscit.2023.101364

**Published:** 2023-11-04

**Authors:** Hubert Provost, Laura M. Drudi, Frank Schwenter, Stéphane Elkouri, Jean-François Blair, Philippe Charbonneau

**Affiliations:** a Faculty of Medicine, Université de Montréal, Montreal, QC, Canada; b Division of Vascular Surgery, Department of Surgery, Centre Hospitalier de l’Université de Montréal, Montréal, QC, Canada; c Department of General Surgery, Centre Hospitalier de l’Université de Montréal, Montréal, QC, Canada; d Aortic Centre, Division of Vascular Surgery, Department of Surgery, Centre Hospitalier de l’Université de Montréal, Montréal, QC, Canada

**Keywords:** Aneurysmectomy, Jejunal aneurysm, Mesenteric aneurysm, Mycotic aneurysm, Superior mesenteric artery aneurysm

## Abstract

Jejunal artery pseudoaneurysms are extremely rare, accounting for <1% of all visceral artery pseudoaneurysms. Fewer than 50 cases were reported in literature during the previous century. This case report describes the case of a 72-year-old man who underwent aneurysmectomy to treat a 21-mm mycotic jejunal artery pseudoaneurysm found in the setting of endocarditis. This pseudoaneurysm was treated with laparotomy, and gentle dissection of the tissues surrounding the pseudoaneurysm was performed before ligation and resection. This allowed for vascular collateral branch preservation, which, thus, avoided concomitant bowel resection. This report highlights the feasibility of this technique.

Patients presenting with a superior mesenteric artery branch aneurysm face a significant risk of death secondary to rupture.^1^ Jejunal artery pseudoaneurysms account for <1% of all visceral pseudoaneurysms and affect mostly older men.^1^ The literature reporting jejunal artery pseudoaneurysms consists of a few case reports and six series.^2^^,^^3^ The main etiology for jejunal pseudoaneurysms is bacterial infection, with *Streptococcus* being the most frequent pathogen, especially in patients diagnosed with endocarditis.^4^^,^^5^ Due to the high risk of rupture, estimated at 30% to 50%, mycotic jejunal artery aneurysms are associated with a high mortality rate.^6^, ^7^, ^8^ An expedited intervention is usually required, which is even more so the case for pseudoaneurysms.^9^ Endovascular embolization of the feeder arteries is not an ideal solution, because the insertion of foreign bodies in an infected field can result in bacterial proliferation. Also, this technique does not allow for direct visualization of bowel tissue, which can subsequently become ischemic. Therefore, open repair is most often considered definitive treatment.^10^ We describe a case of selective ligation and aneurysmectomy of SMA arterial branches without bowel resection as a therapeutic option. The patient provided written informed consent for the report of his case details and imaging studies.

## Case report

A 72-year-old man was transferred to our tertiary care center for treatment of complicated infective endocarditis. His pertinent medical history includes a history of hypertension, atrial fibrillation, type 2 diabetes mellitus, and chronic obstructive pulmonary disease. The patient had been diagnosed with symptomatic atrial flutter in 2013 and underwent cardiac fulguration due to an intolerance to antiarrhythmic drugs. The patient then developed permanent atrial fibrillation and received direct oral anticoagulation therapy. The patient denied alcohol or illicit drug consumption. Three months before being transferred to our hospital, the patient had developed a urinary tract infection (UTI) after elective surgery in September 2021 for transurethral resection of the prostate due to benign prostatic hyperplasia. The UTI was appropriately treated with ciprofloxacin. The patient had undergone catheterization once, did not undergo echocardiography before surgery, and was administrated prophylactic ciprofloxacin. The UTI organism was *Enterococcus faecalis,* with sensitivity to penicillin-G, ciprofloxacin, and nitrofurantoin. The patient had no known bacteremia at this point. He subsequently developed intermittent fever spikes, chills, and fatigue of 3 weeks’ duration. A few weeks later, the patient was diagnosed with endocarditis involving the native mitral valve. No predisposing factors were known, and his diabetes was well controlled. Using transesophageal echocardiography, a floating vegetation measuring 11 mm in length was seen on the mitral valve. Blood cultures were positive for *E. faecalis,* the same organism responsible for the UTI.

**Fig 1:**
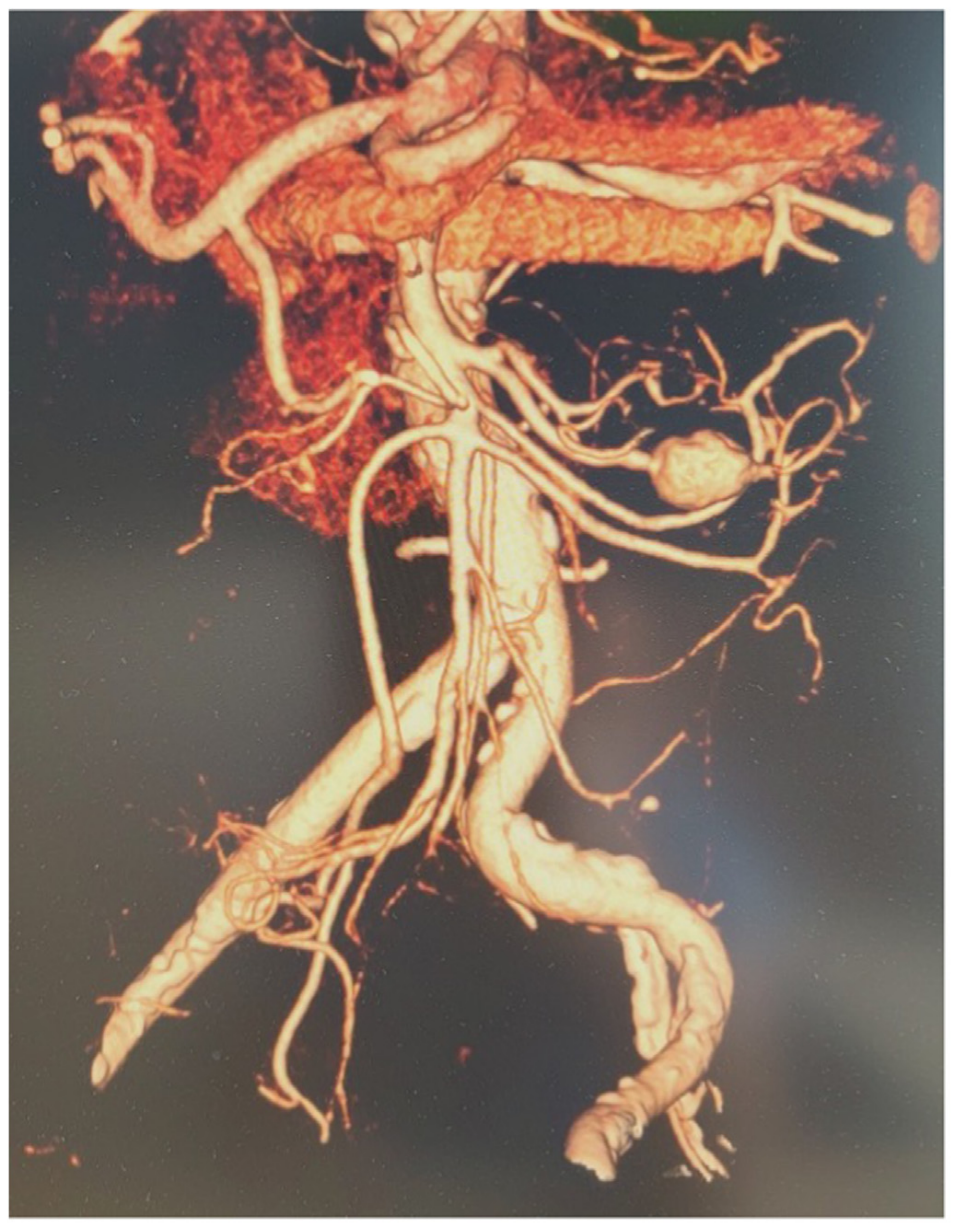
Follow-up computed tomography angiography (CTA) showing a 21-mm jejunal artery pseudoaneurysm with an irregular wall and fat strandings.

On admission, the patient was normotensive with 96% oxygen saturation, neutropenic with a white blood cell count of 3.4 g/L, and his C-reactive protein was high (77 mg/L). He was afebrile and already receiving intravenous ampicillin and ceftriaxone for 8 days before admission. The patient was complaining of lower back pain and paresis of the right lower extremity without any abdominal symptoms. Computed tomography angiography (CTA) revealed spondylodiscitis at the L3-L4 level, an intracerebral abscess without any neurologic symptoms, two infracentimetric intrahepatic pseudoaneurysms, a renal mycotic micro-pseudoaneurysm, and a 9-mm jejunal artery branch pseudoaneurysm. Ten days later, a follow-up CTA showed progression of the jejunal artery pseudoaneurysm from 9 mm to 21 mm, with an irregular wall surrounded by fat stranding (Fig 1). The other previous findings remained unchanged. Considering the fast progression of the pseudoaneurysm, the patient was informed and provided consent for aneurysmectomy with the possibility of bowel resection 2 days after the latest CTA. An endovascular approach was not suitable due to the infectious context and the lack of direct visualization of bowel tissue.

**Fig 2:**
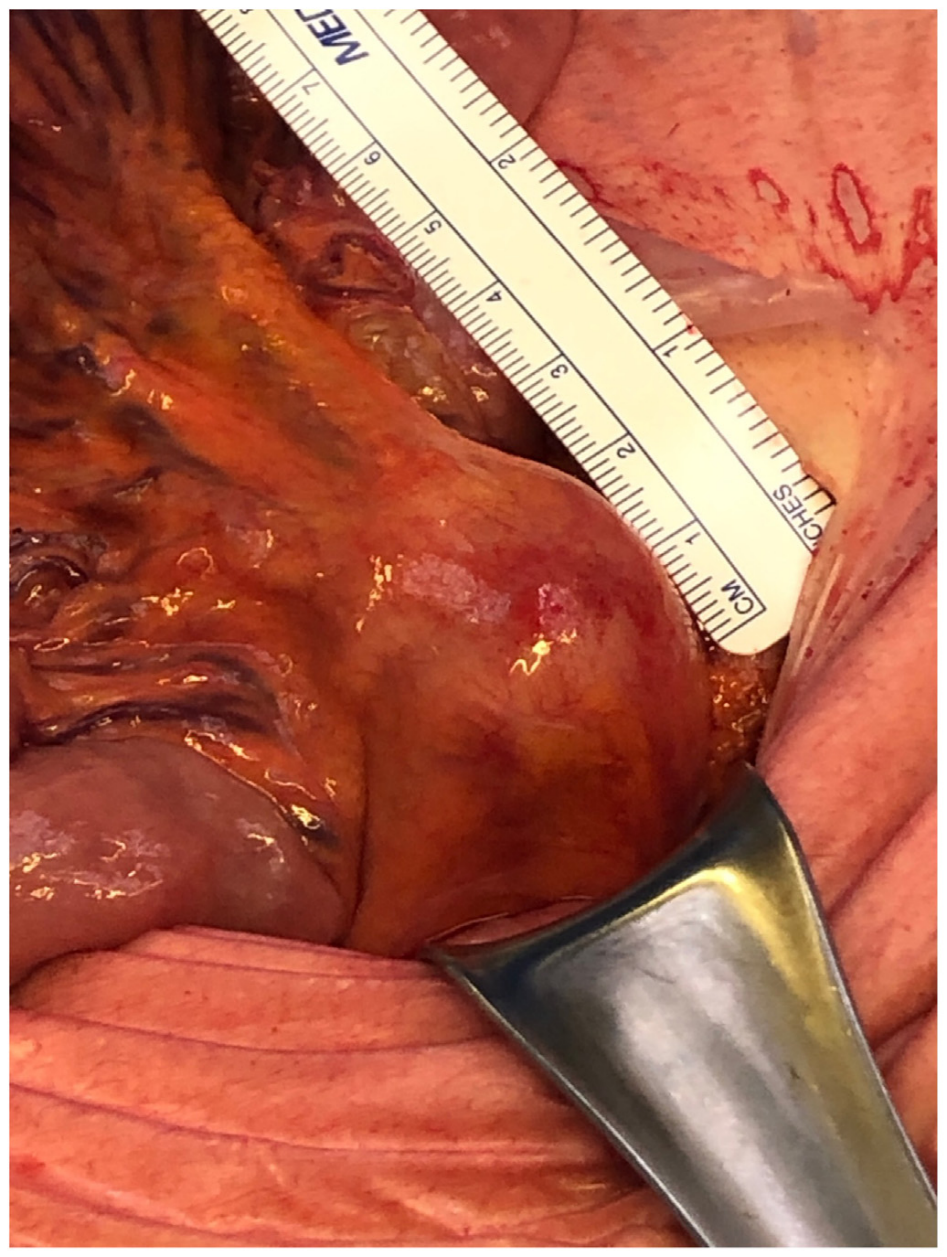
A mycotic aneurysm of a jejunal branch located in small bowel mesentery.

The patient was scheduled for surgery 12 days after admission. He was placed in the dorsal position and under general anesthesia. A standard median laparotomy of 15 cm was performed. The linea alba and parietal peritoneum were opened to expose the intestine. The jejunal artery pseudoaneurysm was easily located in the small bowel mesentery (Fig 2). The inflow and outflow vessels involved in the aneurysmal process were carefully dissected. Approximately eight branches were ligated with 2-0 and 3-0 silk sutures to achieve the aneurysmectomy with preservation of the jejunal artery branches. Arterial collateral pulsatility was visualized after the resection. After waiting 30 minutes, the bowel region at risk of ischemia presented with normal peristalsis and remained well-colored. Doppler ultrasound of the antimesenteric wall of the bowel segment in question was triphasic. Next, the abdomen was closed in the usual fashion, and patient was moved to the recovery room and then the ward. Embolization with coils of the intrahepatic pseudoaneurysms at the IV-B segment was performed 2 weeks later due to progression. Serial imaging showed improvement of the previous findings, including a decrease in size of the vegetation length. However, the patient then underwent mitral valve vegetectomy on January 26, 2022. Transesophageal echocardiography showed no recidivism of the endocarditis. The spondylodiscitis and cerebral abscess were managed conservatively. The patient recovered totally from his right lower extremity neurologic symptoms and was discharged home 1 month later in good clinical condition with intravenous ampicillin and gentamicin. At the last follow-up 2 years after the initial pseudoaneurysm resection, the patient had fully recovered without any residual symptoms.

The pathology report confirmed the diagnosis of a mycotic pseudoaneurysm associated with adventitial fibroinflammatory changes and periarterial fresh thrombus. No microorganisms were found on culture. An increase in IgG4+ plasma cells was noted. However, the global histologic image did not suggest the presence of a IgG4 sclerosing disease, an autoimmune disease in which inflammatory cells cause fibrosis predominantly in the adventitia and potentially cause formation of a pseudoaneurysm.^11^

## Discussion

Infectious causes represent the most frequent etiologies of jejunal artery pseudoaneurysms. The Society for Vascular Surgery guidelines suggests treating aneurysms when the diameter is >2 cm.^10^ Despite their size, mycotic pseudoaneurysms should be treated promptly.

In the present patient, disseminated infectious embolisms from the endocarditis might have been responsible for the multiple pseudoaneurysms, brain abscess, and spondylodiscitis. Often, patients have nonspecific infectious symptoms unless a rupture or structural compression occurs. Considering the high rupture risk of mesenteric pseudoaneurysms, an intervention to exclude the pseudoaneurysm is recommended once diagnosed. Open surgery, including ligation with or without bowel resection, remains the most definitive treatment. Although endovascular treatment has been described, even for ruptured visceral artery pseudoaneurysms,^12^, ^13^, ^14^ open surgery is the preferred option locally, because an endovascular approach requires the availability of an experienced team.^4^^,^^15^^,^^16^ Revascularization with a direct end-to-end anastomosis or the use of an autologous graft could only be conceivable with a larger artery diameter, usually >3 mm. Also, embolization of the intrahepatic pseudoaneurysms at the IV-B segment was the chosen method because of the better accessibility via an endovascular approach compared with open surgery, although the etiology was not optimal for this type of procedure.^17^, ^18^, ^19^, ^20^ According to the Society for Vascular Surgery guidelines, strong evidence suggests that coil embolization of intrahepatic pseudoaneurysms is the treatment of choice.^21^

In the literature, jejunal artery pseudoaneurysms caused by infective endocarditis are rare. A few case reports have described simple ligation and excision of a jejunal branch as an option if the collateral blood flow is adequate and the bowel seems viable.^11^ Only six other mycotic jejunal pseudoaneurysm cases are documented, four involving aneurysmectomy alone, one involving ligation and excision with segmental bowel resection, and one treated conservatively without surgical intervention due to its distal location.^19^^,^^21^, ^22^, ^23^

## Conclusions

Mycotic jejunal branch artery pseudoaneurysms occur rarely. CTA and other imaging modalities are essential to determine the diagnosis and plan an adequate intervention. Open surgery with bowel preservation should be attempted as the first-line treatment when mycotic pseudoaneurysms occur on a small distal branch because it allows for definitive removal of the infected tissue.

## Disclosures

P.C. is a consultant for Cook Medical, Inc. H.P., L.M.D., F.S., S.E., and J.-F.B. have no conflicts of interest.
